# Diagnostic value of preoperative computed tomography-urography combined with inflammatory markers in predicting lymph node metastasis in patients undergoing radical cystectomy

**DOI:** 10.14440/bladder.2025.0012

**Published:** 2025-07-07

**Authors:** Kun Yang, Mingxin Jiang, Tianyu Zhang, Yunpeng Fan, Yongde Xu, Lei Wang, Xi Zhu, Zhengguo Ji, Wei Qiu, Lang Feng, Jun Li, Daoxin Zhang, Gangyue Hao, Yinong Niu

**Affiliations:** 1 Department of Urology, Beijing Friendship Hospital, Capital Medical University, Beijing 100050, China; 2 Institute of Urology, Beijing Municipal Health Commission, Beijing 100050, China

**Keywords:** Bladder cancer, Pelvic lymph node metastasis, Computed tomography-urography, Inflammatory markers

## Abstract

**Background:**

Lymph node metastasis represents a critical prognostic factor in bladder cancer and significantly influences treatment choice and outcomes.

**Objective:**

To evaluate the predictive value of the maximum short-axis diameter of pelvic lymph nodes on preoperative computed tomography-urography (CTU), in combination with inflammatory markers, in the prediction of lymph node metastasis in radical cystectomy (RC) patients.

**Methods:**

A retrospective analysis was conducted on 210 patients who had received CTU within one month before RC at Beijing Friendship Hospital from January 2016 to December 2023. Upon screening, 174 patients were included and assigned into two groups based on postoperative pathology: i.e., lymph node metastasis group (n = 43) and non-metastasis group (n = 131). The neutrophil-to-lymphocyte ratio (NLR), platelet-to-lymphocyte ratio (PLR), and monocyte-to-lymphocyte ratio (MLR) were calculated. The maximum short-axis diameter of a lymph node ≥8 mm was considered indicative of metastasis. Receiver operating characteristic (ROC) curve analysis was performed to assess predictive performance, determine optimal cutoffs, and construct a prediction model using multivariate logistic regression.

**Results:**

Significant differences (*P* < 0.05) were observed between groups in clinical T stage, tumor grade, NLR, PLR, MLR, and CTU lymph node diameter. ROC analysis revealed optimal cutoff values for NLR (3.22), PLR (156.4), and MLR (0.62). Multivariate logistic regression identified clinical T stage, CTU lymph node diameter, MLR, and PLR as independent predictors (*P <* 0.05). The resulting model achieved an area under the curve of 0.847 (95% confidence interval: 0.777 – 0.917).

**Conclusion:**

A nomogram incorporating CTU findings, clinical T stage, MLR, and PLR effectively predicts lymph node metastasis in RC patients. However, further multi-center validation is required before clinical implementation.

## 1. Introduction

Bladder cancer (BC) is the tenth most common cancer across the globe, with a notably higher incidence in men, and urothelial cancer is the predominant pathological type.^1^-^3^ The majority of BC patients present with non-muscle-invasive BC (NMIBC), while approximately 25% of cases are diagnosed with muscle-invasive BC (MIBC). Among MIBC patients, preoperative lymph node metastasis is observed in 25 – 30% of cases. Consequently, radical cystectomy (RC) with pelvic lymph node dissection (PLND) is the standard treatment for MIBC and high-risk NMIBC.^4^

Currently, PLND typically includes the bilateral obturator, internal iliac, and external iliac lymph node groups. Extended PLND involves additional regions, such as bilateral common iliac and sacral lymph nodes. Some surgeons have proposed ultra-extended dissection, expanding the upper limit to the inferior mesenteric artery. However, such an approach increases the risk of surgical complications, including bleeding, nerve injury, ureteral damage, lymphatic fistula, and lymphocele formation.^5^,^6^ Given these risks, accurate preoperative assessment of lymph node involvement is critical for determining the appropriate surgical approach and ensuring personalized treatment for BC patients.

Computed tomography-urography (CTU) is an extensively used imaging technology in clinical practice thanks to its cost-effectiveness and modest equipment requirements. It is commonly employed in the staging, treatment monitoring, and follow-up of BC patients, yielding favorable results. Clinically, the cutoff value for pelvic lymph node metastasis is typically defined as a maximum short-axis diameter of lymph nodes ≥8 mm.^7^ Several studies have reported the predictive sensitivity ranging from 45.5% to 83.0%,^8^,^9^ and the specificity from 63% to 98%. However, the false-negative rate remains high, standing at 25 – 40%, highlighting the limitations of relying solely on CTU for preoperative assessment of lymph node involvement in BC patients.

The role of the inflammatory response in tumors has been increasingly recognized.^10^ Plenty of studies have demonstrated that neutrophils release various inflammatory factors associated with tumor proliferation and metastasis, such as neutrophil elastase and tumor necrosis factor. In contrast, lymphocytes exert anti-tumor effects by inducing cell apoptosis. Macrophages, derived from monocytes, are abundant within the tumor microenvironment and contribute to immune suppression, facilitating tumor development and progression through mechanisms including inflammation promotion and metabolic regulation. Additionally, elevated platelet levels stimulate the synthesis of growth factors, such as platelet-derived growth factor and vascular endothelial growth factor, further promoting tumor progression.^11^

Inflammatory indicators derived from peripheral blood cells, such as the neutrophil-to-lymphocyte ratio (NLR), platelet-to-lymphocyte ratio (PLR), monocyte-to-lymphocyte ratio (MLR), systemic inflammation response index, and systemic immune-inflammation index, have been found to be associated with the risk level and prognosis in BC, prostate cancer, and other malignancies.^12^-^14^ The current investigation focused on three well-established and widely validated systemic inflammatory biomarkers: NLR, PLR, and MLR.

This study retrospectively analyzed patients who had undergone RC and preoperative CTU examination. The predictive value of the maximum short-axis diameter of CTU-detected lymph nodes plus preoperative inflammatory indicators was evaluated. Postoperative pathological findings served as the gold standard to assess the accuracy of this combined approach in determining lymph node metastasis in these patients.

## 2. Materials and methods

### 2.1. Patients

This study retrospectively collected clinical and pathological data from 210 patients who underwent RC at Beijing Friendship Hospital, affiliated with Capital Medical University, between January 2016 and December 2023. All patients had undergone preoperative CTU examination within one month prior to surgery. The inclusion criteria included: (i) patients who underwent RC and PLND, and (ii) patients who had received a CTU examination within one month before surgery. The exclusion criteria involved: (i) Patients pathologically diagnosed as having non-urothelial carcinoma (*n* = 8); (ii) Patients with concurrent malignant tumors of pelvic organs (*n* = 8); (iii) Patients with infectious diseases or those having taken immunosuppressive drugs prior to surgery (*n* = 15); (iv) Patients with incomplete medical records (*n* = 5). Against these criteria, 174 patients were included in the study. Based on the pathological results of pelvic lymph nodes following surgery, patients were divided into a lymph node metastasis group (*n* = 43) and non-metastasis group (*n* = 131). This study was approved by the Ethics Committee of Beijing Friendship Hospital, affiliated with Capital Medical University, Beijing, China.

### 2.2. Observations

Patient data were collected, including age, gender, height, weight, comorbidities, preoperative blood counts (within 1 week before surgery), chemotherapy history, preoperative CTU findings, surgical methods used, urinary diversion types, and postoperative pathological results. Additionally, the NLR, PLR, and MLR were calculated based on the preoperative blood counts. In the CTU examination, the presence of lymph node metastasis was indicated if the maximum short-axis diameter of the pelvic lymph nodes was ≥8 mm.

### 2.3. Statistical analysis

Clinical data were analyzed using the Statistical Package for the Social Sciences 25.0 software package (IBM, USA). Metric data with a normal distribution and homogeneous variance were expressed as mean ± standard deviation, and intergroup comparisons were made using the independent sample *t*-test. Non-normally distributed quantitative data were presented as a median and interquartile range, with intergroup comparisons conducted using the rank-sum test. Count data were given as frequency and proportion, and group comparisons were performed using the Chi-square test. The receiver operating characteristic (ROC) curve, the area under the curve (AUC), and the maximum Youden index were used to estimate sensitivity and specificity, while logistic regression models were applied for univariate and multivariate analyses. A nomogram was constructed based on the indicators with significant differences in multivariate analysis by R 4.1.2 software (A Language and Environment for Statistical Computing, R Core Team, R Foundation for Statistical Computing, Vienna, Austria, https://www.R-project.org). A *P* < 0.05 (two-sided) was considered statistically significant.

## 3. Results

### 3.1. Basic information of the experimental groups

The clinical T stage, tumor grading, maximum short-axis diameter of CTU lymph nodes, and preoperative NLR, PLR, and MLR were significantly higher in patients with lymph node metastasis than in their counterparts without lymph node metastasis (*P* < 0.05). In contrast, there were no significant differences between the two groups in terms of age, gender, body mass index, smoking history, comorbidities, bladder perfusion chemotherapy history, chemotherapy history, surgical methods, urinary diversion types, maximum tumor diameter, tumor number, and the number of PLND. These results are presented in Table 1.

**Table 1:** Basic information of the lymph node metastasis group (pN+) and the non-metastasis group (pN−)

Variable	Overall (*n*=174)	pN+ (*n*=43)	pN− (*n*=131)	*P*-value
Age (year)	67.9±8.8	66.6±8.8	68.3±8.8	0.252
Gender (%)				
Male	158 (90.8)	40 (93.0)	118 (90.1)	0.562
Female	16 (9.2)	3 (7.0)	13 (9.9)	
BMI (kg/m^2^)	24.6±3.3	24.9±3.9	24.5±3.1	0.463
Smoke (%)				
Yes	62 (35.6)	14 (32.6)	48 (36.6)	0.628
No	112 (64.4)	29 (67.4)	83 (63.4)	
Hypertension (%)				
Yes	83 (47.7)	20 (46.5)	63 (48.1)	0.857
No	91 (52.3)	23 (53.5)	68 (51.9)	
Diabetes (%)				
Yes	47 (27.0)	10 (23.3)	37 (28.2)	0.523
No	127 (73.0)	33 (76.7)	94 (71.8)	
Intravesical chemotherapy (%)				
Yes	33 (19.0)	7 (16.3)	26 (19.8)	0.605
No	141 (81.0)	36 (83.7)	105 (80.2)	
Chemotherapy history (%)				
Yes	28 (16.1)	10 (23.3)	18 (13.7)	0.141
No	146 (83.9)	33 (76.7)	113 (86.3)	
Surgical approach (%)				
Open RC	52 (29.9)	11 (25.6)	41 (31.3)	0.477
Laparoscopic RC	122 (70.1)	41 (74.4)	90 (68.7)	
Urinary diversion (%)				
Cutaneous ureterostomy	88 (50.6)	21 (48.8)	67 (51.1)	0.210
Bricker	72 (41.4)	21 (48.8)	51 (38.9)	
Neobladder	14 (8.0)	1 (2.3)	13 (9.9)	
Clinical T stage (%)				
<T2	64 (36.8)	8 (18.6)	56 (42.7)	0.004*
≥T2	110 (63.2)	35 (81.4)	75 (57.3)	
Tumor size (%)				
<3 cm	74 (42.5)	15 (34.9)	59 (45.0)	0.243
≥3 cm	100 (57.5)	28 (65.1)	72 (55.0)	
Solitary tumor (%)				
Yes	131 (75.3)	30 (69.8)	101 (77.1)	0.333
No	43 (24.7)	13 (30.2)	30 (22.9)	
Tumor grade (%)				
Low	19 (10.9)	1 (2.3)	18 (13.7)	0.046*
High	155 (89.1)	42 (97.7)	113 (86.3)	
NLR	2.92 (2.05, 4.48)	3.85 (2.51, 6.97)	2.69 (1.79, 3.69)	<0.001*
PLR	142.0 (104.7, 202.9)	191.1 (140.7, 248.7)	133.6 (96.7, 192.4)	<0.001*
MLR	0.30 (0.20, 0.47)	0.47 (0.28, 0.83)	0.27 (0.19, 0.42)	<0.001*
CTU lymph node				
≥8 mm	48 (27.6)	22 (51.2)	26 (19.8)	<0.001*
<8 mm	126 (72.4)	21 (48.8)	105 (80.2)	
Number of PLND	15 (10, 19)	14 (8, 19)	15 (10, 19)	0.265

Note:

* *P*<0.05.

Abbreviations: BMI: Body mass index; CTU: Computed tomography-urography; MLR: Monocyte-to-lymphocyte ratio; NLR: Neutrophil-to-lymphocyte ratio; PLND: Pelvic lymph node dissection; PLR: Platelet-to-lymphocyte ratio; RC: Radical cystectomy.

### 3.2. Predictive value and cutoff values of neutrophil-to-lymphocyte ratio, platelet-to-lymphocyte ratio, and monocyte-to-lymphocyte ratio in predicting lymph node metastasis

**Figure 1:**
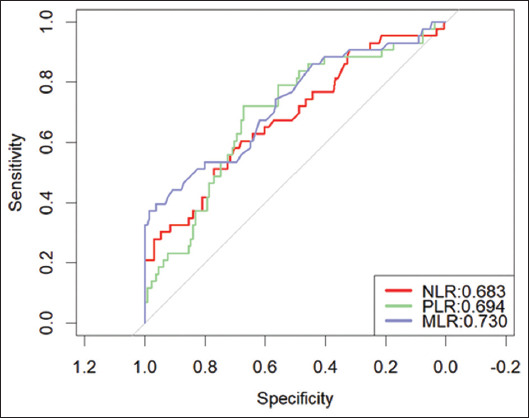
Receiver operating characteristic analysis of neutrophil-to-lymphocyte ratio, platelet-to-lymphocyte ratio, and monocyte-to-lymphocyte ratio

The ROC curve was used for statistical analysis, and the AUC for predicting lymph node metastasis using NLR, PLR, and MLR was 0.683 (95% confidence interval [CI]: 0.587 – 0.779), 0.694 (95% CI: 0.603 – 0.786), and 0.730 (95% CI: 0.636 – 0.824), respectively. The sensitivity and specificity of these ratios were 59.5% and 67.9%, 71.4% and 67.2%, 38.1% and 93.9%, respectively. According to the Youden index, the optimal cutoff values for the three ratios were 3.22, 156.4, and 0.62, respectively. These results are listed in Figure 1 and Table 2.]

**Table 2:** Predictive value of NLR, PLR, and MLR

Variable	AUC	Cutoff value	Youden index	95% CI	Sensitivity (%)	Specificity (%)	*p*-value
NLR	0.683	3.22	0.274	0.587 – 0.779	59.5	67.9	<0.001*
PLR	0.694	156.40	0.386	0.603 – 0.786	71.4	67.2	<0.001*
MLR	0.730	0.62	0.343	0.636 – 0.824	38.1	93.9	<0.001*

Note:

* *P*<0.05. Abbreviations: AUC: Area under the curve; CI: Confidence interval; NLR: Neutrophil-to-lymphocyte ratio; PLR: Platelet-to-lymphocyte ratio; MLR: Monocyte-to-lymphocyte ratio

### 3.3. Multivariate logistic regression analysis of factors associated with lymph node metastasis

On the basis of the results in Table 2, NLR ≥3.22, PLR ≥156.4, and MLR ≥0.343 were converted into binary variables, and multivariate logistic regression analysis was performed on the patients in the lymph node metastasis group. The results showed that clinical T stage, PLR, MLR, and maximum short-axis diameter of CTU lymph nodes were independent risk factors predictive of pathological lymph node metastasis (*P* < 0.05). However, multivariate regression analysis revealed no statistical significance with tumor grades and NLR. These results are shown in Table 3.

**Table 3:** Multivariate logistic regression analysis of factors associated with lymph node metastasis

Variable	OR	95% CI	*P*-value
Clinical T stage	3.196	1.136 – 8.990	0.028*
Tumor grade	2.573	0.280 – 23.656	0.404
NLR	0.943	0.321 – 2.774	0.916
PLR	4.241	1.502 – 11.977	0.006*
MLR	11.259	2.948 – 43.006	<0.001*
CTU lymph node	4.441	1.786 – 11.043	0.001*

Note:

* *P*<0.05.

Abbreviations: CI: Confidence interval; CTU: Computed tomography-urography; MLR: Monocyte-to-lymphocyte ratio; NLR: Neutrophil-to-lymphocyte ratio; OR: Odds ratio; PLR: Platelet-to-lymphocyte ratio.

### 3.4. Nomogram validation of the results

**Figure 2:**
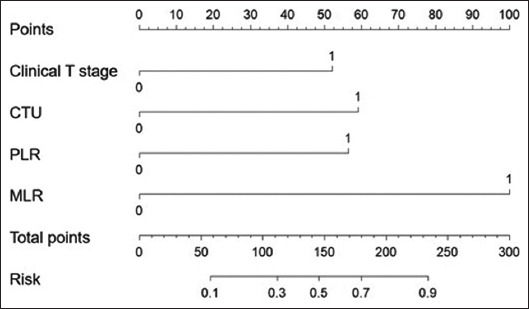
Nomogram model for predicting lymph node metastasis in bladder cancer patients Abbreviations: CTU: Computed tomography-urography; MLR: Monocyte-to-lymphocyte ratio; PLR: Platelet-to-lymphocyte ratio.

**Figure 3:**
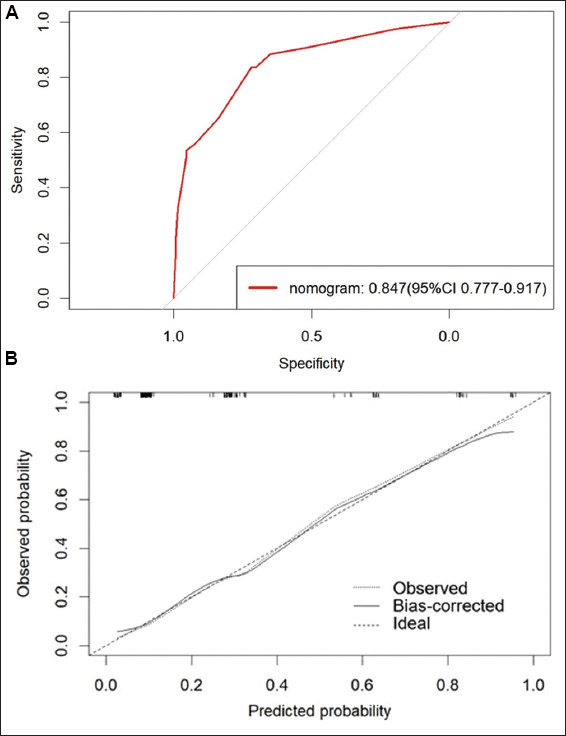
Receiver operating characteristic analysis (A) and calibration curve (B) of the nomogram model Abbreviation: CI: Confidence interval.

Based on the results of multivariate logistic regression analysis, a nomogram was developed (Figure 2). Clinical T stage, CTU, PLR, and MLR were included in the construction of the nomogram, with MLR playing the most important role. The model demonstrated an AUC of 0.847 (95% CI: 0.777 – 0.917), indicating excellent discriminative performance. Furthermore, the calibration curve closely aligned with the ideal reference line, reflecting strong agreement between the predicted and observed probabilities. These findings suggest that the model has both high discriminability and robust diagnostic accuracy, as illustrated in Figure 3.

## 4. Discussion

Lymph node metastasis is a critical factor influencing recurrence and progression in BC patients. Approximately 80% of patients with lymph node metastasis suffer from postoperative recurrence or progression, while the recurrence rate for patients without nodal involvement is roughly 30%. Moreover, the 5-year survival rate in BC patients without lymph node metastasis is about 60%, whereas it drops to 15 – 31% for those with lymph node involvement.^1^,^3^ PLND is a key component of RC for patients with MIBC or high-risk NMIBC. However, debate regarding the optimal extent of PLND still lingers. The primary surgical approaches include standard, extended, and ultra-extended lymph node dissections. Leissner *et al*.^15^ reported that approximately 8% of BC patients had presacral lymph node involvement. Similarly, Steven and Poulsen,^16^ in a study of 336 RC with PLND patients, found that about 34% of positive lymph nodes were located outside the boundaries of standard lymph node dissection, potentially leading to residual disease if a standard lymph node dissection fails to identify these lymph nodes.

Extending the scope of lymph node dissection is critical for accurate pathological N staging and the removal of residual metastasis. Nonetheless, evidence remains mixed. A study involving 933 patients found that, in BC patients with pT0-pT2 tumors, extended PLND did not significantly improve disease-free survival or tumor-related survival compared to standard PLND.^17^ Additionally, other studies have shown that extended lymph node dissection significantly increases surgical time and postoperative complications.^18^ Given these findings, it is crucial to accurately assess lymph node involvement preoperatively to determine the optimal extent of lymph node dissection in BC patients.

The sensitivity and specificity of conventional pelvic computed tomography (CT) and magnetic resonance imaging (MRI) for detecting pelvic lymph node metastasis remain low.^8^,^19^ Notably, the metastatic lymph nodes identified by imaging account for only 27.7% of pathologically-confirmed findings. In a study involving 184 patients, Li *et al*.^9^ reported that 82 patients were diagnosed with lymph node metastasis based on CT or MRI. However, pathological verification revealed that only 51 of these 82 patients had positive metastatic lymph nodes, resulting in a sensitivity of 83.0% and a specificity of 64.3%. In the present study, pelvic lymph nodes with a maximum short-axis diameter ≥8 mm on CTU were considered indicative of metastatic involvement, yielding a predictive sensitivity of 51.2% and a specificity of 80.2%, which aligns with previous findings.

Previous studies have identified NLR, PLR, and MLR as prognostic markers for risk stratification across various malignant tumors.^13^,^20^-^24^ For example, a study involving 127 patients with gastrointestinal stromal tumors demonstrated that those with NLR ≥4.16, PLR ≥257.1, and MLR ≥0.54 had significantly lower recurrence-free survival compared to other patients, with MLR showing the strongest predictive value.^25^ Likewise, in a cohort of 510 patients who underwent transurethral resection of bladder tumors, Napolitano *et al*.^26^ found that 340 patients (66.6%) were pathologically diagnosed as having BC. They observed that MLR levels were significantly higher in patients with BC than in those with benign pathology (*P* = 0.043), suggesting elevated MLR as a potential predictive marker for BC. Furthermore, a recent study from Tongji University reported that MLR >0.54, NLR >4.10, and PLR >164.63 were associated with reduced overall survival and recurrence-free survival in patients receiving RC.^27^ Notably, the AUC of MLR was significantly larger than those of NLR and PLR. Additionally, other studies have indicated that NLR and PLR were closely linked to the postoperative lymph node stage and the number of lymph node metastases in gastric cancer patients.^28^

The present study showed that patients with lymph node metastasis had significantly higher NLR, PLR, and MLR compared to those without metastasis. Multivariate logistic regression analysis identified PLR and MLR as independent risk factors for lymph node metastasis in BC patients. Additionally, ROC curve analysis determined the cutoff values for PLR and MLR to be 156.4 and 0.62, respectively, consistent with findings from previous studies. Although NLR exhibited a significant difference in univariate analysis, it did not reach statistical significance with multivariate analysis. This discrepancy may be attributed to two factors: first, the retrospective nature of the study limited the ability to assess the specific clinical conditions of patients during surgery, resulting in the exclusion of all patients with neutrophil counts higher than the normal reference value; and second, the inclusion of patients with a history of chemotherapy or intravesical chemotherapy may have influenced their NLR levels.

The roles of MLR and PLR in BC are still not fully understood. Monocytes and lymphocytes each contribute differently to the disease progression. Monocytes can differentiate into macrophages within the tumor microenvironment, where they secrete immunosuppressive factors, such as interleukin-10 and tumor growth factor-beta, inhibiting anti-tumor immune responses and promoting immune escape.^29^,^30^ Furthermore, during tumor progression, monocytes can express immune checkpoint molecules, such as programmed cell death protein 1 and cytotoxic T-lymphocyte protein 4, facilitating the development of immune tolerance and supporting tumor cell survival. In contrast, lymphocytes play a pivotal role in the host immune defense against tumors by inducing the apoptosis of tumor cells, inhibiting their growth and migration, and mediating cytotoxic effects.^31^ Additionally, platelets have been shown to promote tumor angiogenesis through secreting angiogenic factors, such as vascular endothelial growth factor and platelet-derived growth factor, providing essential nutrients for tumor growth and expansion.^32^,^33^

Previous studies have developed nomogram models to predict lymph node metastasis in BC patients, primarily using imaging and pathological data.^34^,^35^ However, few studies have incorporated inflammatory indicators, such as MLR and PLR, into these models. The current study integrated MLR and PLR with preoperative clinical stages and CTU results to establish a clinical prediction model that demonstrated strong predictive performance.

However, this study is subject to some limitations. First, its retrospective design and single-center, regional patient cohort might introduce selection bias. Second, only the most prevalent and readily accessible inflammatory markers derived from blood count results were included, while other potentially relevant biomarkers were excluded from the analysis. Further research, including comparative studies and basic experimental validation, is required to explore their potential role. In this study cohort, 89.1% of patients had high-grade tumors, and multivariate logistic regression analysis revealed no significant difference in outcomes in terms of tumor grade. As a result, tumor grade was not included in the prediction model. To strengthen the generalizability and reliability of these findings, prospective studies incorporating larger, multi-center sample sizes are warranted.

## 5. Conclusion

A nomogram integrating CTU, clinical T stage, MLR, and PLR was developed to predict lymph node metastasis in BC patients. However, the retrospective single-center design and limited cohort (*n* = 174) may compromise statistical robustness, potentially introducing selection bias and overfitting risks. Therefore, prospective multi-center studies with larger, more diverse cohorts are imperative prior to its clinical application.

## Data Availability

The data used and analyzed during the current study are available from the corresponding author on reasonable request.
